# Artificial Intelligence Models for the Mass Loss of Copper-Based Alloys under Cavitation

**DOI:** 10.3390/ma15196695

**Published:** 2022-09-27

**Authors:** Cristian Ștefan Dumitriu, Alina Bărbulescu

**Affiliations:** 1 Doctoral School, Technical University of Civil Engineering Bucharest, 124, Lacul Tei Bd., 020396 Bucharest, Romania; 2 Department of Civil Engineering, Transilvania University of Brașov, 5, Turnului Street, 900152 Brașov, Romania

**Keywords:** mass loss, cavitation, ultrasound, corrosion–erosion, artificial intelligence (AI)

## Abstract

Cavitation is a physical process that produces different negative effects on the components working in conditions where it acts. One is the materials’ mass loss by corrosion–erosion when it is introduced into fluids under cavitation. This research aims at modeling the mass variation of three samples (copper, brass, and bronze) in a cavitation field produced by ultrasound in water, using four artificial intelligence methods—SVR, GRNN, GEP, and RBF networks. Utilizing six goodness-of-fit indicators (R^2^, MAE, RMSE, MAPE, CV, correlation between the recorded and computed values), it is shown that the best results are provided by GRNN, followed by SVR. The novelty of the approach resides in the experimental data collection and analysis.

## 1. Introduction

An ultrasound signal passing through a liquid gives birth to cavitation, a phenomenon defined by the cyclic apparition, increase, and collapse of the bubbles of vapors formed in the liquid [1]. During these cycles, a voltage is induced near the cavitation zone’s boundaries [2,3,4]. Cavitation is an exogenous process accompanied by discontinuities in the liquid’s state, which appear when the pressure drops under critical limits [5]. Emulsification, noise, vibrations, sonoluminescence, unpassivation, and corrosion are some of its multiple effects [6,7].

Corrosion is a complex process affecting most of the metallic materials used in industry. Electrochemical corrosion occurs at the contact of a metal with an electrolyte (for example, seawater) by charge transfer through the interface [8]. Oliphant [9] classified the copper alloys’ corrosion types into pitting, rosace corrosion, induced microbiological corrosion, erosion–corrosion, and corrosion induced by welding flux. Regardless of the corrosion type, the cavitation effects are determined by the material and environmental characteristics [10].

Analyzing and monitoring the process apparition and the damages produced on different installations and components (such as ballast installation, bilge systems, propellers) working in cavitation conditions are essential for ensuring their correct functioning and the workers’ safety. Therefore, erosion–corrosion and materials’ mass loss as effects produced by cavitation have become topics of interest for many scientists [11,12,13,14,15].

Several investigations on cavitation corrosion, mainly from the viewpoint of the driving mechanism, have been performed by different authors [16,17,18]. Wharton and Stokes [15] studied the corrosion process of bronzes with Ni and Al. Basumatary et al. [11] found that the Ni-Al bronze has a significantly better corrosion resistance than the duplex stainless steel in cavitation (both materials are usually used for building propellers). It was also shown [12,19] that the presence of Sn in different bronzes increases their resistance to high-pressure vapors of seawater. The alloys’ mechanical properties were improved by adding Ni in bronzes with Fe and Al. The resulting materials had an increased resistance to corrosion in cavitation media.

The results of Kumar et al. [20] show that the increase in the lead content of brass from 1 to 3.4 wt.% increases the corrosion resistance and improves the stability of the alloy in both chloride and sulfate media due to the passive film formation. The literature [21,22] specifies that finely processed surfaces are more resistant than those with high roughness. It was also shown that the damage of the materials introduced in a cavitation field is more intense than that in the same medium without cavitation [23].

It is known that there is a direct relationship between the material’s resistance and its mass loss. The lower the resistance is, the higher the mass loss is, since the links between the atoms and molecules in the material can be easily broken when the resistance is low. Nevertheless, despite the extensive study of the corrosion mechanism, only a few researchers (Schüssler and Exner [13], Fortes-Patella et al. [24], Dumitriu [25], Dumitriu and Barbulescu [26]) have analyzed the copper-based alloy samples’ mass loss in liquids under cavitation from a quantitative viewpoint. None of them [13,24,25,26] approached the subject with artificial intelligence tools (AI).

AI algorithms provide solutions to a large variety of problems. Approaches such as Support Vector Machine (SVR) [27], Artificial Neural Networks (ANNs) [28], General Regression Neural Networks (GRNN) [29], Gene Expression Programming (GEP) [30,31,32], adaptive neuro-fuzzy interference (ANFIS) [33], and decision tree (DT) [34] have been utilized in engineering and finance for solving optimization problems. Radial basis networks (RBFs), multilayer perceptrons (MLPs), artificial regression trees, and Random Forest were successfully employed to predict the mass loss in Electro-Discharge Machining [35]. The reliability of some components from different industrial case studies has been forecast by four artificial intelligence methods [36]. GEP, AdaBoost, and XGBoost were applied to evaluate the high-strength concrete properties in [37,38].

In the above context, the present article aims at modeling the mass loss of samples of brass, bronze, and copper (for comparison) in a cavitation field produced in an experimental setup built by our team for such purposes. Four AI algorithms are employed for this aim (SVM, GRNN, RBF, and GEP) and the best results (with respect to six indicators) are presented. It is shown that all algorithms performed well on the studied series, but the best was GRNN. In comparison with the classical parametric regression, this approach has the advantage that it does not rely on restrictive hypotheses on the data sets, and any condition does not constrain the residuals.

The novelty of the research resides in the following:

- The experimental setup was designed by our team.
- The experiments on materials mass loss have been performed in the cavitation field produced by ultrasound; this approach is important for knowing the mass-loss behavior of some materials used in naval construction.
- Based on our knowledge, the analysis of the mass loss of copper-based alloys in the ultrasound cavitation field was not extensively performed.
- Modeling the mass loss of such alloys in the cavitation field has not been performed using AI methods.
- Knowing the mass loss is important for predicting the behavior of different components built using such materials; a good model can be used to obtain a forecast that can be utilized for predicting the replacement periods in an integrated reliability study.

## 2. Materials and Methods

### 2.1. Experiments

**Figure 1:**
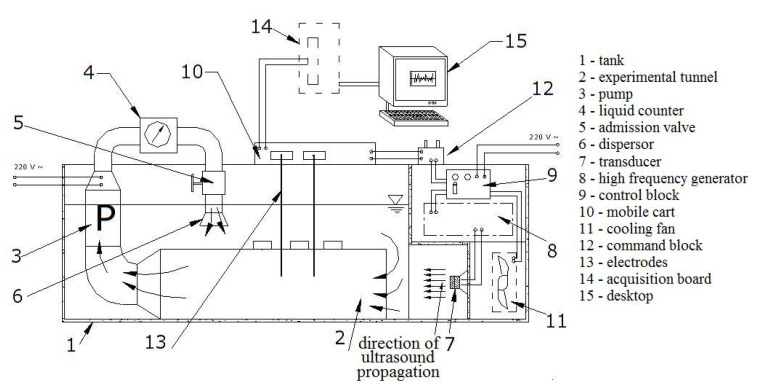
Experimental plant for the cavitation study [39].

The experimental plant built for performing the study of ultrasound cavitation and the materials’ mass loss is presented in Figure 1.

Its main parts are as follows [3,39]:

- The tank (1) containing the liquid subject to the ultrasound cavitation;
- The high-frequency ultrasound generator (8), designed to work at 220 V, 18 kHz, and three power levels;
- The piezoceramic transducer (7) that produces cavitation entering into oscillation as a response to the high-frequency signal received from the generator;
- The control panel (command block) (12) from where the ultrasound generator’s working power is selected;
- The cooler (11) that is used to maintain a constant temperature of the liquid;
- The measurement electrodes (13), which are utilized only in the experiments related to capturing the signal induced in the cavitation field;
- The acquisition data unit (14), used only in the experiments related to electrical signals induced by ultrasound cavitation to collect the signals.

For experiments in the circulating liquid medium—not discussed in this article—the pump (3) is switched on. The following conditions have been met in the experiments whose results are presented here.

The studied samples had the following compositions:

- Cu containing small percentages of Fe, Sn, and Zn (0.0395%, 0.0446%, and 0.0747%, respectively);
- A brass with 2.75% Pb, 38.45% Zn besides Cu (57.95 %);
- A bronze containing Zn, Pb, and Sn (4.07%, 4.40%, and 6.4%, respectively) besides Cu.

The samples had a hexagonal shape, with a side of about 1.5 cm. They were suspended by rigid plastic wires inside the tank at a distance of about 20 cm from the transducer.

The ultrasound generator worked at 180 W, and the water temperature was maintained at 20 °C. The samples were kept in saline water under cavitation produced by ultrasound for 1320 min, cleaned, and weighted every 20 min. The composition of the seawater used for all the experiments is the following: pH = 7, 22.17 g/L NaCl, 0.051 mg/L Fe, 0.0033 mg/L Ni, 0.31 g/L ${\mathrm{SO}}_{4}^{2-}$, total water hardness—6.27 meq/L.

The experiments were performed in triplicate.

**Figure 2:**
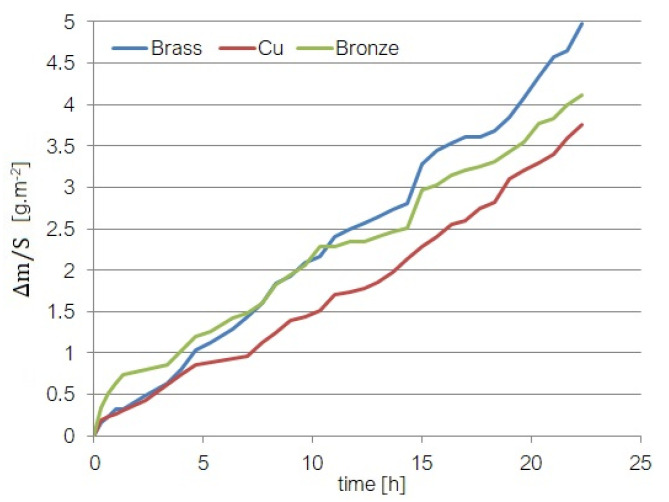
Data series.

To estimate the ultrasound effect on the samples from a quantitative point of view, the mass variation (computed by the difference between the sample’s mass at the experiment’s beginning and the mass at the moment *t*) on the surface (S) was recorded for the modeling stage. The data series are represented in Figure 2.

The mass loss variation can be explained by a complex mechanism (electrochemical and erosion–corrosion). Since the material is introduced in seawater with a high concentration of NaCl, two opposite processes appear: (a) passivation leading to the formation of Cu oxides deposited on the sample surface; (b) removal of these oxides due to the ultrasound action. In the first phase, the sample mass increases, while in the second one, it decreases, explaining the oscillations observed in the chart.

Moreover, as a cavitation effect, the material’s microcrystalline structure is weakened by the erosion–corrosion that breaks the atomic links. Consequently, the material’s resistance decreases, resulting in microfissures and cracks. These fissures become new sites where the material is broken and detached from the sample. Therefore, the material mass loss is more accelerated when it is no longer maintained in the cavitation field, increasing the mass loss rate. The material loss is not uniform because it is composed of different elements with various structures and resistances to cavitation.

### 2.2. Modeling Methodology for the Weight Loss

The AI methods utilized in this work for modeling the mass loss of the materials in the described conditions are SVR, GRNN, GEP, and RBF.

SVR [40,41,42] belongs to the class of supervised learning algorithms characterized by high performances correlated with a low computational cost in solving regression and classification problems. Using training sets composed of *n* pairs $\;\left(x_{k},y_{k}\right),$ $\mathrm{where}\;\;x_{k}$ (*k =* $\bar{1,n}$) are the vectors of known features from a domain D in the *m*-dimensional space,$\;R^{m}$ ($x_{k}\in D\subset R^{m})$ $\;\mathrm{and}\;y_{k}\in R$ are the target values, the algorithm output is a function *f* based on which unknown $\hat{\;y_{k}}$ are provided when $x_{k}$ are given. The model function *f*, in the linear case, is

(1) $$f\left(x\right)=\left(u,x\right)+b,\;u\in R^{m},\;x\in D,$$

where (.,.) is the inner product in *D*, and *b* is a real constant.

SVR produces prediction functions mapped on a set S⊂D of vectors support [27] and *f* should minimize the objective function

(2) $$\frac{1}{2}{\left\|u\right\|}^{2}+C\overset{n}{\underset{k=1}{\sum }}L(\;y_{k},f\left(\;x_{k}\right)),$$

where ‖ ‖ is the norm in $L^{2}$, C∈(0,∞), *L*(.,.)  is a ε-insensitive loss function [40,41].

The maximum deviation between the target and the *f* function’s values must be less than ε on the training set. The number of support vectors is controlled by ε,  whereas *C* ensures a balance between the model function’s flatness and the bias from the ε−tube [27,41].

The nonlinear problems are transformed into linear through some mappings, Ψ, defined using different kernels K:D×D→R, where K(u,v) is the inner product of Ψ(u)  and Ψ(v) in Ψ(D).

One of the most important aspects of SVR modeling is choosing the kernel type. The most used kernels are RBF, linear, sigmoid, and polynomial. Their choice significantly influences the model quality. Therefore, for our study, we performed experiments with all these types of kernels. Since the best results were obtained utilizing a linear kernel, we report them in the article.

Another aspect is related to the time necessary to perform the experiments. SVR is the worst compared with GEP, GRNN, and RBF. It takes a few minutes to run the algorithm, compared with a few seconds for the other three methods. The time consumed increases when the number of the kernel’s parameters to be estimated increases and the step size in the grid search for each parameter decreases.

After choosing the linear kernel, the step size in the grid search was selected to be 0.1 (to maintain a balance between the time and parameters’ quality) and the parameter C was searched in the interval [0, 50,000].

The number of predictors should also be selected for performing the algorithm. Theoretically, the user should choose the number of predictors based on his experience or the series characteristics. After performing experiments with different predictors, and given that the mass loss depends on the mass value before running the experiment, the number of predictors was chosen to be one, which is the lag 1 variable (the sample’s absolute mass loss at the previous moment).

For modeling, the data series was divided into two parts—the first for training and the second for testing. Different ratios were tested. Here, we report the best results obtained for the ratio training:test = 70:30 (70% of the data series for training the model and the rest for the testing). A 4-fold cross-validation was used in the training–prediction process. The optimization criterion was to minimize the total error.

For details of SVR, the reader may see the articles of Vapnik [40] and Smola [41].

**Figure 3:**
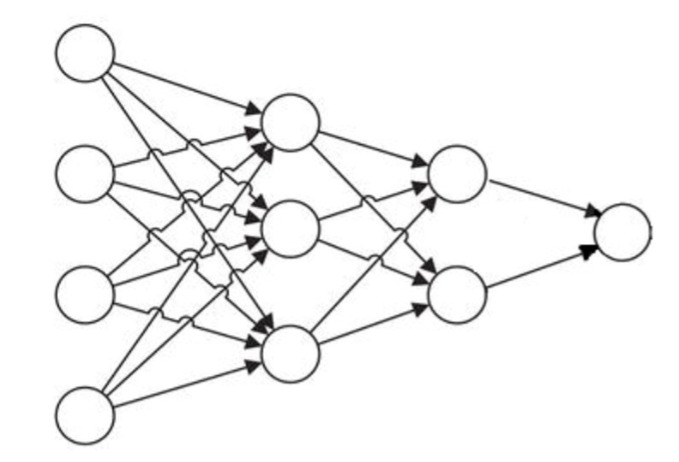
The scheme of a GRNN.

GRNNs (Figure 3) are ANNs with four layers—input, hidden, summation, and output. These feedforward networks process the information in successive order from one layer to another without feedback [43].

The neurons in the hidden layers symbolize different patterns. They calculate the distance (usually Euclidean) between each input data and a center point and apply an activation function to these distances.

Each training sample, $X_{i}$, is utilized as the mean of a Gaussian distribution

(3) $$Y\left(X\right)=\frac{\sum_{i=1}^{n}Y_{i}\exp(-D_{i}^{2}/\left(2\sigma^{2}\right))}{\sum_{i=1}^{n}\exp(-D_{i}^{2}/\left(2\sigma^{2}\right))},\;$$

where

(4) $$D_{i}^{2}={\left(X-X_{i}\right)}^{T}\left(X-X_{i}\right)\;$$

and $D_{i}$ is the distance between the training sample and the prediction point.

$D_{i}$ is a measure of how well each training sample can represent the position of prediction, X. When $D_{i}$ is small, $\exp(-D_{i}^{2}/\left(2\sigma^{2}\right))\;$ is big. When $D_{i}=0,$ $\exp(-D_{i}^{2}/\left(2\sigma^{2}\right))=1,\;$ and the evaluation point is best represented by the training sample. A high $D_{i}$ produces a small $\exp(-D_{i}^{2}/\left(2\sigma^{2}\right))$; as a consequence, the contribution of the other training samples to the prediction is relatively small. If σ is big, the possible representation of the evaluation point by the training sample is possible for a wider range of X, whereas when σ is small, the representation is limited to a narrow range of X [44]. Since the influence radius of each neuron is controlled by σ, this parameter must be found in the training process to optimize the network’s performance.

The third layer is formed by the S- and D-summation neurons, which sum up the information received from the previous layer (weighted by the first one).

The GRNN is described by the equation [45]

(5) $$Y=W_{ho}\varphi \left(XW_{ih}+b_{I}\right)+b_{o}\;$$

where the symbols have the following meanings:

***X***—the vector containing the network input;

***Y***—the output vector;

φ—the activation function;

$W_{ih}$—the matrix of the weights of the input in the hidden layer;

$W_{ho}$—the matrix containing the weights of the results of the hidden layer;

$b_{I}\left(b_{o}\right)$—the error vector corresponding to the first (hidden layer).

In this study, to select the optimum σ−s,  the employed algorithm was the conjugate gradient, and the search was performed in the interval [0.0001; 10]. The convergence tolerances were 10^−8^—absolute and 10^−4^—relative, and the upper limit of the number of iterations (without improvement, respectively) was 5000 (1000, respectively). As for SVR, the best results are reported here. They were obtained when running the algorithm with a ratio of 70:30 between the training and test sets.

RBF belongs to the feedforward ANNs and is built of three layers—input, hidden, and output. The first layer’s output is obtained by computing the distance between its input and the second layer’s centers. The hidden layer has as output the weighted values of the output of the first layer. The center is a vector of the hidden layer’s neurons. An activation function (usually Gaussian) is associated with the neurons from the second layer. This function has a spread parameter that controls its behavior.

The output of an RBF network can be described by [46]

(6) $$\hat{y_{k}}=\overset{J}{\underset{j=1}{\sum }}w_{jk}\varphi \left(\|x-c_{j}\|\right)+\beta_{k},\;j=\bar{1,J},\;k\bar{1,K},$$

with

$\hat{y_{k}}\;$—the *k*th neuron’s output;

**x**—the input data vector;

**c***_j_* —the *j*th neuron center vector;

*J* (*K*)—the number of neurons belonging to the second (third) layer;

$w_{jk}$—the weight corresponding to the *j*th and *k*th neuron and output;

‖‖—the Euclidean norm;

$\beta_{k}$—the bias corresponding to the *k*th neuron’s output;

φ—the RBF function:

(7) $$\varphi \left(\left\|x-c_{j}\right\|\right)=\exp\left(-\alpha_{j}\|x-c_{j}\|{}^{2}\right);$$

$\alpha_{j}$—the spread parameter (radius) that controls the *j*th neurons’ spread.

For the network’s performance evaluation, MSE or RMSE are usually utilized.

The RBF training is performed to minimize the objective function, which is the sum of square errors, defined by

(8) $$SSE=\overset{K}{\underset{k=1}{\sum }}{\left(y_{k}-\hat{y_{k}}\right)}^{2}$$

where $y_{k}$ is the recorded value and $\hat{y_{k}}$ is the result after running RBF [47].

In RBF networks, choosing the neurons’ number in the hidden layer affects the network’s complexity and its generalizing capability. If this number is insufficient, the network cannot learn the data adequately. If this number is very high, it may result in overfitting or limited generalization capacity [48,49].

It was also shown that the centers’ detection (in the second layer) significantly influences the performance of the RBF network [49]. Therefore, one of the main tasks when training the network is determining the best center positions.

The RBF network training must also include the optimization of the spread parameters of each neuron and the selection of the weights between the second and third layers. Therefore, in the training process, the number of neurons, the centers in the second layer, the spread parameters, and the weights must be appropriately selected. To achieve this goal, the network’s training was performed by an orthogonal forward algorithm proposed by Chen et al. [50] that employs tunable center vector nodes for building the RBF function and minimizes the leave-one-out procedure. The algorithm does not need to impose a stop criterion. The ridge regression was utilized for the weights’ computation.

For tuning the neurons’ parameters, the size of the population was fixed to 200, maximum number of generations—20, maximum generations flat—5, and maximum boosting tolerance—10^−4^. The used networks’ parameters were maximum number of neurons—100, minimum (maximum) radius = 0.01 (400), and absolute tolerance—10^−6^.

The reader may refer to [47,51,52,53] for more details on RBF networks.

Genetic Algorithms (GAs) are evolutionary techniques based on the principle of Darwinian selection, operating on populations of individuals, successively created by choosing the best individuals based on their fitness. The selected individuals are combined using specific operations to give birth to new generations. The steps of this procedure are (a) random initialization of the population, (b) fitness assignment, (c) individuals selection, (d) crossover and mutation. The algorithm is run until the stop criterion is met [54]. Random mutations do not permit GAs to remain captured in a locally optimal region.

**Figure 4:**
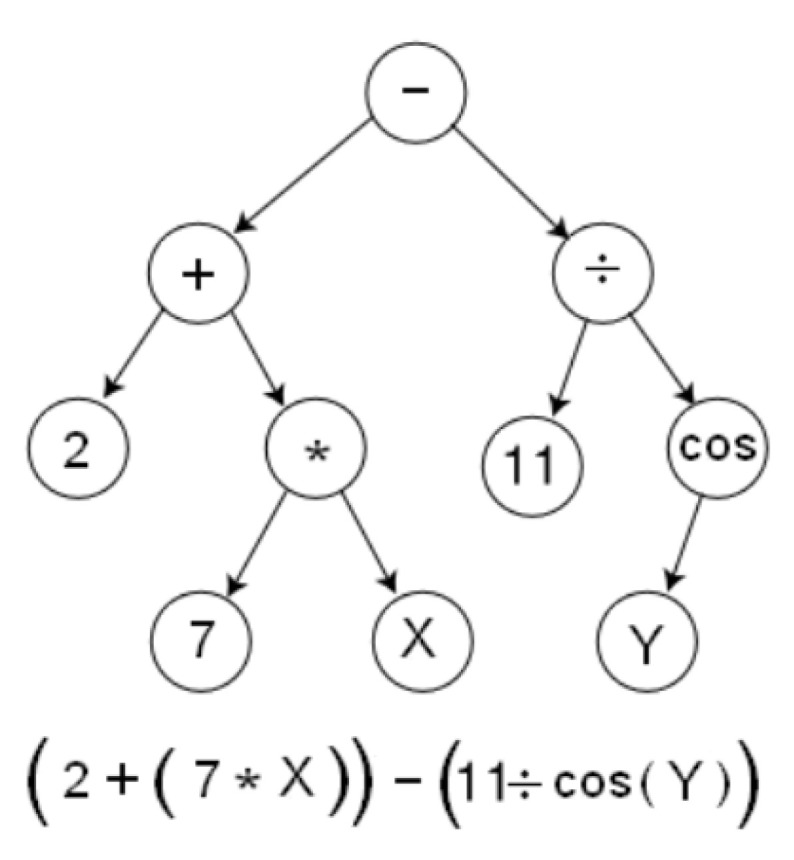
The parse tree and the encoded expression.

Introduced in 1988 by Koza [55], Genetic Programming (GP) belongs to the same class of evolutionary techniques [56] and differs from others by the information’s representation as programs inspired by nature mechanisms [57]. GP provides the solution to the problem at hand, enabling the computer to search for it and deliver it in the form of parse trees whose nodes but the terminal ones contain operators. The terminal nodes contain constants and variables so the expressions can be quickly evaluated and evolved. For example, the tree in Figure 4 presents the parse tree that encodes the expression 2 + (7 * X) − (11/cos(Y)).

Gene Expression Programming (GEP), proposed by Ferreira in 2001 [58], incorporates features from GAs (such as linear chromosomes of fixed length) and GP (the structure of a tree with different shapes and sizes).

A chromosome is a string consisting of elements belonging to (a) and (b) that is mapped into a tree that has, as a correspondent, a unique mathematical formula [59]. It contains at least one gene, which is formed, at its turn, by several symbols (fixed) and has a head (that contains constants, variables, and functions) and a tail (containing terminal symbols–constants and variables). When a minimum of two genes are present, they are connected by a linking function to generate the solution to the problem at hand.

For solving a problem using GEP, one must specify (a) the set of functions, (b) the terminal set (that contains constants and variables), (c) the fitness function, (d) the control parameters, and (e) the stop condition.

To start, GEP randomly generates a population of chromosomes evaluated with respect to a fitness function defined by the user. Then, the best individuals are chosen, using the roulette-wheel selection criterion, for producing the next generation using genetic operations (mutation, transposition, crossover). Still, elitism is also applied: the individual with the highest fitness in each generation goes without modifications to the next generation. The number of genes and the linking function must be specified before running the algorithm. In all cases, the main point is that the solution to a problem is represented by individuals evolving and improving from one generation to the next. The process continues until the stop criterion is met.

It should be mentioned that the expression with the simplest form is preferred among the expressions with the same performances.

For details on GEP, the reader may see [58,60].

In the experiments, we performed 50 independent runs for each setup.

In this article, the following settings have been used to run the algorithm:

- Number of genes per chromosome—4.
- Length of the head of a gene—8.
- Number of constant per gene—10.
- The size of the population—50 individuals.
- The maximum number of generations (and without improvement)—2000 (1000).
- The fitness function—MSE, and the hit tolerance—0.01.
- The functions used to build the final expression—{+, −, *, /, sqrt}.
- The linking function was the addition.
- The algorithm was allowed to do algebraic simplification.
- The mutation (and inversion) rate—0.44 (0.1).
- The transposition rate—0.1.
- The one-point (two-point and gene) recombination rate—0.3 (0.3 and 0.1).
- For the experimental reasons explained above, the regressor in the model was the lag 1 variable, $X_{t-1},\;$ to predict $X_{t}$.
- The obtained models are compared with respect to the MSE values obtained on the original data set; in the following, we report only the best model (i.e., the model with the smallest mean standard error) found in all 50 runs of the algorithm.
- Experiments were performed using different ratios between the Training and Test sets, such as 80:20 or 90:10; the best results are presented in this article, which were obtained for the Training set formed by 70% of the data series, and the Test set formed by the rest of the series’ values.

To compare the algorithms’ performances, the following indicators were used: the proportion of variance explained by the model (R^2^), the coefficient of variation (CV), the correlation between actual and predicted (r_ap_), the mean absolute and mean absolute percentage error (MAE and MAPE), and the root mean squared error (RMSE).

The DTREG software [61] was employed for running the algorithms.

**Figure 5:**
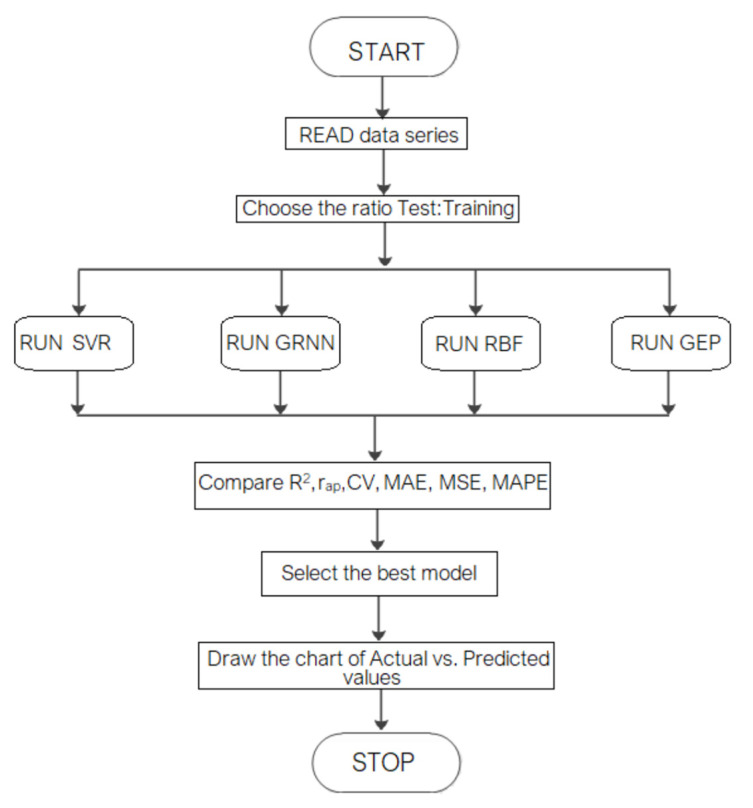
The flowchart of the modeling and estimation process.

The flowchart of the study is presented in Figure 5.

## 3. Results

### 3.1. Results on the Cu Sample Mass Loss

The goodness of fit indicators for modeling the copper mass loss by the methods presented in Methodology are given in Table 1—on the Training (columns 2–5) and Test (columns 6–10) sets. The R^2^ and r_ap_ are close to 1, indicating a very good concordance between the computed and recorded values on both sets. The highest values correspond to the GRNN algorithm on both sets.

The SVR with a linear kernel gave the best results. During search, the number of evaluated points was 191, the minim error = 0.002853, and the parameters’ values—the tube-width ε = 0.00003787 and C = 0.15714469. Twenty-two support vectors were utilized during the process. In GRNN, the optimal sigma corresponding to the regressor was 0.0006164, and 1156 evaluations were performed. In the RBF network, the number of neurons was found 2, and the radius was between 0.01 and 5.8808.

In the GEP model, the complexity of the model after simplification was 9, and the fitness function was evaluated 110,150 times. The expression generated was

(9) $$y_{t}=0.5094507y_{t-1}/\left(1-y_{t-1}\right)+0.0004684,\;t\geq \;2$$

Since the lower the coefficient of variation is, the better the fit is, the best algorithm is GRNN (CV = 0.0141 and CV = 0.0137 on the Training and Test set, respectively). The lowest MAPE (77 × 10^−7^ and 1.1612, respectively), RMSE, and MAE correspond to GRNN as well. The r_ap_ values are almost equal in all methods on the Test set, while on the Training one, the one corresponding to GRNN (0.9997) is higher than the others (equal to 0.9967).

The second best algorithm on the Training set is GEP, considering the first four indicators, and SVR, in terms of MAE and MAPE. On the Test set, the second-best algorithm is GEP, with respect to all indicators but MAE.

Table 2 contains the actual, computed values; the errors; and the absolute percentage error (denoted by % error) from the Test procedure. The % error on the Test set in GRNN is between 0.056 and 2.729 (the amplitude 2.673); in SVR, it is in the interval [0.085, 2.869] (amplitude = 2.784); in GEP, it is between 0.179 and 2.669 (amplitude = 2.490); and in RBF, in the range 0.098–3.154 (amplitude = 2.956).

**Figure 6:**
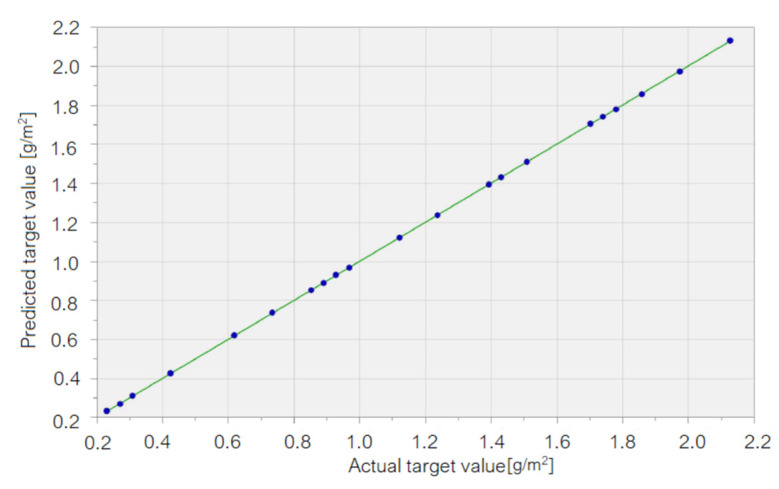
The computed values (called “predicted target values”) vs. the recorded ones (called “actual target values”) in the GRNN model for the Cu sample.

Figure 6 displays the chart of the computed values in GRNN (named “Predicted target values”) vs. the recorded ones (named “Actual target values”). Since the dots are aligned along the line representing the perfect fit between the computed and recorded values, the chart confirms that the GRNN is the most appropriate to model the mass loss of the Cu sample.

### 3.2. Results on the Brass Sample Mass Loss

The results of modeling the brass sample’s mass loss by different AI methods are presented in Table 3. Columns 2–5 contain the results on the Training set, while the last four columns are filled in with the results on the Test set.

When running the SVR algorithm, the best results were obtained with a linear kernel, and the parameters C = 2.26564144 and ε = 0.00001459. During the search, 196 points were checked, 23 support vectors were utilized, and the minimum error computed was 0.007092. In GRNN, the optimum sigma was 0.00217, and 2652 evaluations were performed. In the RBF network, the number of neurons was found to be 4, and the radius was between 0.01 and 5.8804.

In the GEP model, the complexity of the model after simplification was 30, whereas the fitness function was estimated at 118,300 times. The expression generated was

(10) $$y_{t}=-46.262975y_{t-1}^{3}+y_{t-1}/\left(y_{t-1}+2.9923668\right)+y_{t-1},\;t\geq \;2.$$

On the Training set, GRNN performed the best, having the lowest RMSE, MAE, MAPE, and CV. On the Test set, SVR performed the best based on all indicators but MAE. In terms of MAE, the best algorithm was GRNN.

A comparison between the output of the Training and Test sets show the following:

- Point of view of R^2^, r_ap_, RMSE, and MAE for all algorithms provided the best results on the Training sets;
- Point of view of CV, all but GRNN gave the best results on the Test sets;
- From the MAPE viewpoint, the best results were provided by GRNN on the Test set and by SVR on the Test set.

Table 4 contains the absolute percentage error in the AI models on the Test sets.

The amplitudes (difference between the maximum and minimum % errors) are 4.103 (SVR), 5.403 (GRNN), 7.493 (GEP), and 5.506 (RBF), confirming the SVR and GRNN’s performances.

**Figure 7:**
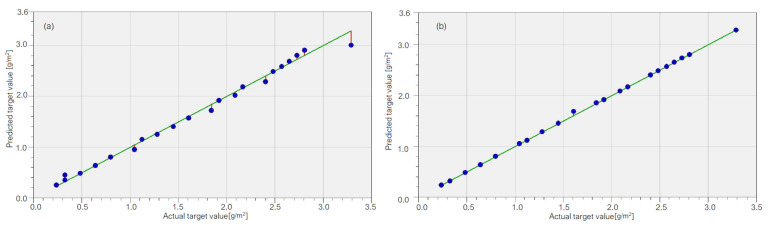
The computed values (called “predicted target values”) vs. the recorded ones (called “actual target values”) in (**a**) the SVR model and (**b**) the GRNN model for the brass sample.

Figure 7 presents the chart of computed values (“predicted target values”) vs. the “actual target values” (recorded) in the SVR and GRNN models on the Training set. Since the closer the points (whose coordinates are the pairs (recorded values, computed values)) are to the first bisector of the axes of coordinates (the green line), the better the fit is, one may remark that the best model is GRNN (Figure 7b).

Overall, the best model is GRNN because there are no significant mismatches between the parameters’ values on the Test set for both methods, but on the Training set, MAPE is much lower for GRNN than for SVR; overall, the best model is GRNN.

### 3.3. Results on the Bronze Sample Mass Loss

When running the SVR algorithm, the best results were obtained with a linear kernel, and the parameters *C* = 1.1930123 and ε = 0.04402791. One hundred seventy points were checked when running the algorithm, the minimum error found was 0.005817, and thirteen support vectors were employed.

In GRNN, the optimum sigma was 0.1143176, and 850 evaluations were performed. In the RBF network, the number of neurons was found to be 2, and the radius was between 6.7531 and 9.0718.

In the GEP model, the complexity of the model after simplification was 13, and the number of fitness function evaluations was 114,400. The expression generated was

(11) $$y_{t}=6.028799y_{t-1}^{4}+y_{t-1}-9.6586234y_{t-1}^{3},\;t\geq \;2.$$

Table 5 shows the indicators’ values after running the algorithms for the bronze.

On both sets—Training and Test—the highest R^2^ and r_ap_ and the lowest CV, RMSE, MAE, and MAPE are issued from the GRNN model, which proved to have the highest performances among the four algorithms. One may notice that the MAPEs for the GRNN are at least 1.670 (1.338) times higher than the corresponding MAPEs of the competitors on the Training (Test) set.

The absolute % errors on the Test set are also small—between 0.146 and 0.510. Table 6 contains the values of the errors and the absolute % errors in the GRNN model on the Test set. The values of the last parameter vary between 0.501 and 5.150.

**Figure 8:**
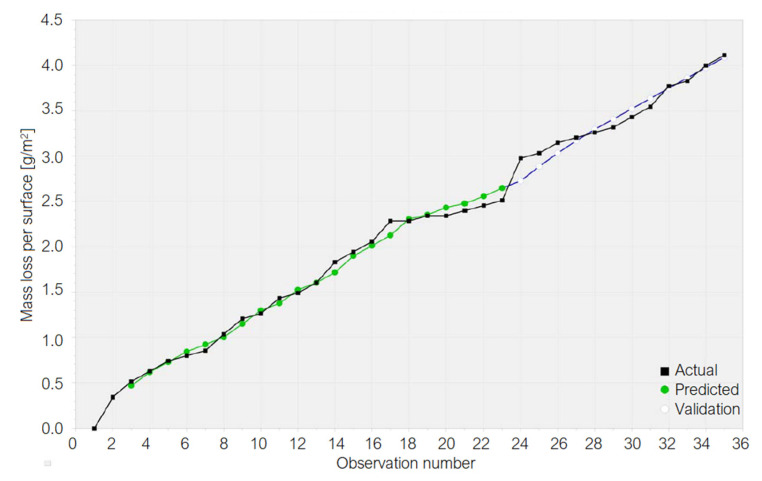
The series of the recorded values (called “Actual”), values computed in the Training procedure (called “Predicted”), and those calculated in the Test procedure (called “Validation”) in the GRNN model for the Cu sample.

Figure 8 displays the recorded values (called “Actual”), those computed during the Training procedure (called “Predicted”), and those in the Test procedure (called “Validation”) in the GRNN algorithm. The shapes of the “Predicted” and “Validation” series are close to the “Actual” series, confirming the previous findings on the model quality.

## 4. Discussion

In [23,25,26], the authors proved that the power at which the ultrasound generator works is another factor affecting the mass loss, given that the cavitation effect is intensified at a higher power level compared with a lower one. Still, the best AI algorithm (with the same regressors) for modeling the mass loss in the experiments, performed when the generator worked at 80 W and 120 W, was GRNN. For the sake of brevity, the results are not reproduced here.

A complementary approach is to find the absolute mass loss variations as a function of time. The best nonlinear regression models obtained for the same data series are provided in the following.

The equation of the mass loss of the Cu sample is

(12) $$\frac{\Delta m_{t}}{S}=0.1684+0.1079t+0.0023t^{2}\;\left(R^{2}=0.9968\right)$$

that for the brass sample is

(13) $$\frac{\Delta m_{t}}{S}=0.0732+0.1922t+0.0008t^{2}\left(R^{2}=0.9957\right)$$

and that for the bronze sample is

(14) $$\frac{\Delta m_{t}}{S}=0.3384+0.1741t-0.0004t^{2}\;\left(R^{2}=0.9910\right)$$

Despite that these models are easier to be obtained, and $R^{2}\;$ is close to one in all cases, their main drawback is that the residuals do not satisfy all the conditions (normality, homoscedasticity, the correlation absence) necessary to validate the model from a statistical viewpoint. Moreover, if a model is not validated, it cannot be used for forecasting for different reasons. For example, the residuals’ autocorrelation leads to the errors’ propagation and the increase in their amplitude. The heteroskedasticity may involve the existence of many subseries with different behaviors, making the forecast based on the entire series inconsistent.

**Figure 9:**
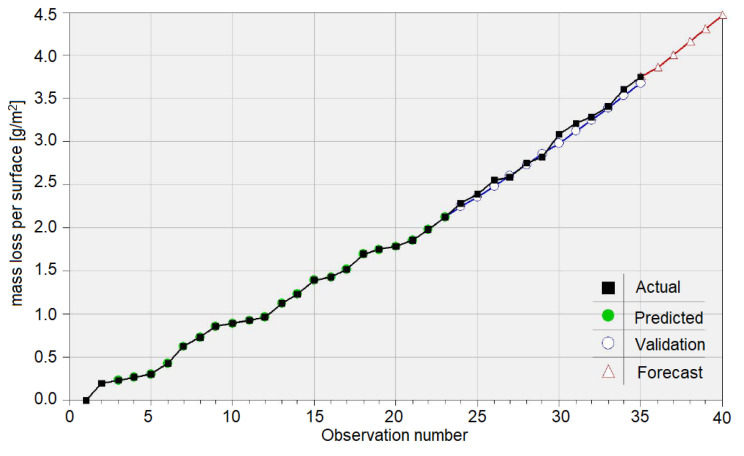
GRNN model with two regressors (lag 1 variable and time) for the mass loss per surface of the Cu sample.

An essential difference between the AI models and those from Equations (12)–(14) is that the regressor is the lag 1 variable in the first case and the time in last ones. Knowing that the mass loss at a certain moment depends on the sample mass and the experimental time, a natural idea is to extend the research by considering both regressors. Since this is an extensive work to be presented in detail in a future article, we introduce only the GRNN model for copper. Figure 9 shows the output of modeling the absolute mass loss of the copper sample, where “Actual”, “Predicted”, and “Validation” have the same significance as in Figure 8. “Forecast” represents the series of the future values computed based on the trained model.

The goodness-of-fit indicators in the model are as follows, respectively:

- On the Training set: R^2^ = 99.935%, CV = 0.0151, MAE = 0.0033, RMSE = 0.016, r_ap_ = 0.99972, and MAPE = 10^−8^,
- On the Test set: R^2^ = 98.369%, CV = 0.0198, MAE=0.0504, RMSE= 0.0590, r_ap_ = 0.9964, and MAPE = 1.6719.

The model with two regressors is better than that with one regressor on the Training (Test) set point of view of all indicators (but R^2^). The absolute % error in the Test set varies between 0.403 and 3.468; so, the amplitude is smaller than in the model with one regressor.

The gain of using both regressors should be also evaluated against the use of only one regressor (lag 1 variable) to determine the essential influence factor.

## 5. Conclusions

The paper presents the finding on the absolute mass loss per surface of Cu, brass, and bronze samples in a cavitation field produced by a high-frequency generator in seawater. It was shown that all four artificial intelligence approaches provide good modeling results for all the samples, but the best in terms of the majority of the goodness of fit indicators proved to be GRNN for Cu and bronze, followed by SVR for brass.

For the future research, four directions are considered. The first one is modeling the mass loss in time and per surface in the same liquid (and other liquids) in the cavitation absence and to compare the results of the present study with the new ones. The second direction is to model the mass loss of the material using multiple regressors, such as, for example, the concentrations of the elements in the samples, time, and the environmental conditions (temperature, seawater concentration). The third direction is to develop a model that relates the material mass loss to the voltage induced by cavitation at different generator’s power level. The fourth one is to model the dependence between the material resistance and the mass loss during the experimental stages. The results of these studies will contribute to a better understanding of the corrosion–erosion process of the studied materials in cavitation field.

## Figures and Tables

**Table 1:** The goodness of fit test for the Cu sample mass loss in the cavitation field.

Set	Training				Test			
Indicator	SVR	GRNN	RBF	GEP	SVR	GRNN	RBF	GEP
R^2^ (%)	99.290	99.943	99.315	99.339	98.923	99.206	99.166	99.206
CV	0.0500	0.0141	0.0491	0.0482	0.0161	0.0137	0.0141	0.0138
r_ap_	0.9967	0.9997	0.9967	0.9967	0.9963	0.9962	0.9960	0.9962
RMSE	0.0528	0.0149	0.0519	0.0510	0.0479	0.0410	0.0422	0.0412
MAE	0.0422	0.0031	0.0453	0.0448	0.0402	0.0345	0.0350	0.0353
MAPE	6.5800	77 × 10^−7^	7.3097	7.0305	1.3421	1.1612	1.1957	1.1935

**Table 2:** Actual and computed values in GRNN on the Test set on the Cu sample.

Actual	Computed	Error	% Error
2.2840	2.2734	0.0106	0.465
2.4002	2.4015	−0.0013	0.056
2.5550	2.5258	0.0292	1.145
2.5938	2.6500	−0.0562	2.169
2.7486	2.7756	−0.0270	0.982
2.8260	2.9031	−0.0771	2.729
3.0970	3.0329	0.0641	2.070
3.2131	3.1650	0.0481	1.500
3.2906	3.2994	−0.0088	0.269
3.4067	3.4363	−0.0296	0.869
3.6003	3.5757	0.0246	0.683
3.7551	3.7176	0.0375	0.999

**Table 3:** The goodness of fit test for the brass sample mass loss in the cavitation field.

Set	Training				Test			
Indicator	SVR	GRNN	RBF	GEP	SVR	GRNN	RBF	GEP
R^2^ (%)	99.231	99.969	99.619	99.555	94.501	94.197	93.574	90.628
CV	0.0563	0.0112	0.0397	0.0429	0.0291	0.0299	0.0315	0.0380
r_ap_	0.9964	0.9999	0.9981	0.9978	0.9773	0.9724	0.9715	0.9662
RMSE	0.0854	0.0170	0.0602	0.0651	0.1173	0.1205	0.1268	0.1532
MAE	0.0580	0.0048	0.0502	0.0546	0.1004	0.0981	0.1022	0.1240
MAPE	6.2502	0.2009	6.9911	6.9481	2.463	2.4912	2.6025	3.1965

**Table 4:** Absolute % error in AI models on the Test set.

SVR	GRNN	RBF	GEP
2.368	3.458	2.621	3.012
1.676	1.757	1.119	0.745
0.573	0.134	0.522	1.394
3.012	3.831	4.461	5.676
4.474	5.537	6.106	7.506
3.797	4.986	5.482	6.951
1.234	2.463	2.884	4.348
0.986	0.244	0.600	2.040
2.932	1.723	1.420	0.013
1.366	0.149	0.117	1.535
4.676	3.520	3.296	1.942

**Table 5:** The goodness of fit test for the bronze sample mass loss in the cavitation field.

Set	Training				Test			
Indicator	SVR	GRNN	RBF	GEP	SVR	GRNN	RBF	GEP
R^2^(%)	98.629	99.066	98.730	98.849	92.271	98.686	92.029	91.312
CV	0.0594	0.0490	0.0572	0.5746	0.0294	0.0220	0.0299	0.0312
r_ap_	0.9932	0.9957	0.9938	0.9944	0.9768	0.9834	0.9795	0.9836
RMSE	0.0887	0.0732	0.0854	0.0813	0.1020	0.0762	0.1036	0.1081
MAE	0.0676	0.0426	0.0665	0.0613	0.0789	0.0601	0.0794	0.0873
MAPE	5.0594	2.4270	5.0261	4.0533	2.4427	1.8253	2.4556	2.6488

**Table 6:** Recorded and computed values in the GRNN on the Test set for the bronze sample.

Recorded	Computed	Error	Absolute % Error
2.9724	2.8194	0.1530	5.150
3.0296	2.9639	0.0657	2.168
3.1439	3.1282	0.0157	0.501
3.2011	3.1495	0.0516	1.613
3.2582	3.2993	−0.0409	1.260
3.3154	3.4634	−0.1470	4.463
3.4297	3.4944	−0.0647	1.886
3.5441	3.6393	−0.0952	2.688
3.7727	3.8036	−0.0309	0.818
3.8298	3.8248	0.0050	0.133
4.0013	3.9745	0.0268	0.671
4.1156	4.1385	−0.0229	0.556

## Data Availability

Not applicable.
